# The Influence of Self-reported Exercise and Sleep Duration and Quality on Next-day Cortisol Rhythms in Older Adults

**DOI:** 10.1093/geroni/igaf122.3858

**Published:** 2025-12-31

**Authors:** Taylor Fein, Orfeu Buxton, Soomi Lee

**Affiliations:** The Pennsylvania State University, University Park, Pennsylvania, United States; The Pennsylvania State University, University Park, Pennsylvania, United States; The Pennsylvania State University, University Park, Pennsylvania, United States

## Abstract

Cortisol shows high day-to-day variability and reflects physiological stress and functioning of the hypothalamic-pituitary-adrenal (HPA) axis. This study examined how sleep and exercise interact with cortisol rhythms, specifically the cortisol awakening response (CAR) and diurnal cortisol slope (DCS). Data from the National Study of Daily Experiences (NSDE 3; 2016-2020), with 699 CAR observations from 279 participants (Mage=63, SDage=10) and 546 DCS observations from 227 participants. For four consecutive days, participants provided four saliva cortisol samples daily and reported time engaging in moderate-to-vigorous exercise, and sleep duration and quality. Multilevel models adjusted for trait (e.g., age, sex, race, education, BMI, smoking, drinking, medication) and state-like (e.g., wake time, prior day CAR/DCS, weekend/weekday) covariates examined independent and interactive effects of sleep and exercise on cortisol. There were no independent associations, however interactive associations were found. Cross-level interactions emerged between within-person prior-day exercise hours and between-person sleep hours (B = 0.06, SE = 0.03, p = 0.035). Simple slope analyses showed that among adults who slept longer than others on average (≥8.2 hours), controlling for within-person sleep, exercising more than usual on the prior day was associated with a flatter DCS the following day. This effect was driven by higher than usual bedtime cortisol, suggesting residual physiological stress and fatigue. Second, a within-person interaction between prior-day exercise and previous-night sleep quality was observed (B=-0.18, SE = 0.08, p = 0.021). On days following more exercise and better sleep quality, DCS had a steeper decline than usual. These findings suggest that daily exercise may influence cortisol rhythm depending on both habitual and nightly sleep.

